# What are the nursing competencies related to antimicrobial stewardship and how they have been assessed? Results from an integrative rapid review

**DOI:** 10.1186/s13756-022-01189-6

**Published:** 2022-12-06

**Authors:** Matteo Danielis, Domenico Regano, Anna Castaldo, Maria Mongardi, Tania Buttiron Webber

**Affiliations:** 1 ANIPIO, Società Scientifica Nazionale degli Infermieri Specialisti del Rischio Infettivo - National Association of Nurses for the Prevention of Hospital Infections, Bologna, Italy; 2 School of Nursing, Department of Medical Sciences, University of Udine, Viale Ungheria 20, 33100 Udine, Italy; 3 IRCCS Azienda Ospedaliero-Universitaria di Bologna, Via Albertoni 15, 40138 Bologna, Italy; 4 IRCCS Fondazione Don Gnocchi, Via Capecelatro, 66, 20148 Milano, Italy; 5 Infectious Diseases Division, Diagnostics and Public Health Department, University of Verona, P. le L.A. Scuro 10, 37134 Verona, Italy; 6 Medical Oncology, Galliera Hospital, Mura delle Cappuccine 14, 16128 Genoa, Italy

**Keywords:** Antimicrobial stewardship, Nurses, Nursing competency, Rapid review

## Abstract

**Background:**

Antimicrobial resistance issues, and the consequent demand for antimicrobial stewardship (AMS) programs, need to be investigated urgently and clearly. Considering the large amount of time nurses spend at patients’ bedside, the aim of the present study was to examine recent literature on nursing competency in AMS.

**Methods:**

Drawing from Tricco and colleagues’ seven-stage process, a rapid review was performed. MEDLINE, CINAHL and EMBASE databased were searched from December 1st, 2019 until December 31st, 2021. Article screening and study selection were conducted independently by three reviewers. Data were analyzed narratively and categorized adopting an inductive thematic coding.

**Results:**

Sixteen studies met the inclusion criteria and were included. Publications were mainly authored in USA (n = 4), Australia and New Zealand (n = 4) and Asia (n = 4), followed by Europe (n = 2) and Africa (n = 2). Ten studies were quantitative in design, followed by qualitative (n = 4) and mixed-methods studies (n = 2). Nursing competency in AMS seems to be influenced by a two-dimensional model: on the one hand, internal factors which consisted in knowledge, attitudes and practices and, on the other hand, external aspects which are at environmental level in terms of structures and processes.

**Conclusion:**

This study provided a map of dimensions for researchers and practitioners to consider when planning clinical governance, educational activities, and research programs. Significant opportunities exist for nurses to contribute to practice, education, research, and policy efforts aimed at reducing antimicrobial resistance.

## Background

Antimicrobial resistance (AMR) is considered a global threat whose magnitude has grown to the point that the World Health Organization (WHO) has taken the lead for a coordinated and multisectoral response [1]. The dramatic increase in the incidence of infectious syndromes goes hand-in-hand with health expenditures. At patient level, recent data suggest that there were nearly five million deaths associated with bacterial AMR in 2019 and that, by 2050, 10 million people per year are expected to be killed by AMR [2]. Six pathogens (i.e., *Escherichia coli*, *Staphylococcus aureus*, *Klebsiella pneumoniae*, *Streptococcus pneumoniae*, *Acinetobacter baumannii*, and *Pseudomonas aeruginosa*) account for 73.4% of deaths attributable to bacterial AMR [2]. As to expenditures, AMR not only exhausts more than nine billion euros per year in Europe but also an additional 20 billion dollars in direct healthcare costs in the US [3], with a projection of over 100 trillion US dollars to the worldwide economy by 2050 [4].

Antimicrobial stewardship (AMS) refers to the proper use of antimicrobials–including antibiotics, antivirals, antimalarials, and antifungals–to preserve their future effectiveness [5]. Nurses’ involvement in AMS is widely recommended [6] as they make up most of the healthcare workforce at the patient’s bedside. This provides the theme for several reviews that have been recently developed and that document the role of nurses as antimicrobial stewards, in both generic [6–9] and specific contexts of care (e.g., intensive care unit) [10]. The most recent review on nurse role and nurse contribution to AMS, which was published in 2021, covers the period from 2008 to November 2019. The authors examined 52 papers hghlighting that nurses’ involvement might be increased if they were formally welcomed into the AMS team and given the necessary training and support [8].

The first objective of the WHO Global Action Plan on Antimicrobial Resistance, which was adopted in 2015, prioritizes AMR claiming that the key approach to address the lack of expertise in antimicrobial misuse is to ensure that healthcare workers are educated and trained to develop the necessary competencies [5]. This can be achieved through the standardization of educational sources for AMR based on global evidence and best practices [5]. Limited research on nursing competency in AMS and its impact on patients’ outcomes resulted in the lack of an unambiguous definition of which actors are likely to influence nurses’ competency and involvement in AMS [6]. Moreover, especially in the last two years, the coronavirus disease 2019 (COVID-19) pandemic has had a significant impact on the undertaken AMS activities whose long-term outcomes are not yet known [11]. As critically ill COVID-19 patients suffer more frequent bacterial or fungal healthcare associated infections, an increased use of antimicrobials inevitably occurs. Therefore, identifying the up-to-date evidence is an issue of great relevance and it may help both nurse managers and educators to develop strategies for sustaining and enhancing nurses’ competencies in antimicrobial use and optimization.

A preliminary search aimed to explore available knowledge on nurses’ competency in AMS in the field was conducted by examining the MEDLINE database (via PubMed) and PROSPERO in November 2021. No recently published or ongoing reviews emerged on this specific topic (= nurses’ competency on AMS); meanwhile, a high number of AMS articles regarding stewardship in nursing field were published in the last years. The intent of this rapid review was to examine the extant and current literature on nursing competency in AMS, to develop strategies to enhance nurse’s participation in AMS programs, and to identify the gaps in education that can consequently be closed and guide training and educational programs.

## Methods

### Study design

An integrative rapid review as a knowledge synthesis approach capable of providing timely information [12] has been carried out in January 2022. The field of AMS is rapidly changing. Hence, timely reviews can offer a description of current research to report on modifications at both clinical and organizational levels [13].

## Needs assessment and topic selection

The primary need was to map the most recent data on nurses’ knowledge of AMS with the intent to summarize the level of nursing competence and to highlight the methods and metrics for evaluating it. Thus, the review questions were: Which dimensions have been recently studied in their influence on AMS competency in nurses? How were these aspects measured? According to the WHO competency framework for healthcare workers’ education and training on antimicrobial resistance, competencies are a “combination of knowledge, skills, motives and personal traits”, whose development should help individuals to constantly improve their performance and to work more effectively [5]. For the purpose of this review, competency included level of knowledge, perceptions, beliefs, behaviors, roles, opinions, and attitudes [5].

## Study development

According to the methodological process inspired firstly by Tricco and colleagues in 2017 [13], which was then further developed by Langlois and collegues in 2019 [14], the following seven-stage process was implemented: (1) needs assessment and topic selection; (2) study development; (3) literature search; (4) screening and study selection; (5) data extraction; (6) risk-of-bias assessment; and (7) knowledge synthesis. In addition, the Preferred Reporting Items for Systematic reviews and Meta-Analysis (PRISMA) guidelines were applied [15]. The review was pre-registered with the international prospective register of systematic reviews (PROSPERO registration number CRD42022311711).

## Literature search

MEDLINE (via PubMed), Cumulative Index to Nursing and Allied Health Literature (CINAHL), and EMBASE databases were searched from December 1st, 2019 until December 31st, 2021. This timeframe was selected in order to capture the most recent trends in the research topic. Furthermore, 2019 was chosen as a year because a similar review (= nurses’ role and contribution to AMS) had already searched up to November 2019 [8]. This research included studies: (a) assessing nursing competency in AMS; (b) using both primary quantitative and qualitative study designs; (c) regarding any clinical setting; and (d) published in English. As in some countries (e.g., USA, Netherlands, Australia) prescribing competencies are directly integrated in nursing curricula, studies on nurse prescribers or nurse practitioners were included, as well as studies on nursing students. In addition, studies with multidisciplinary team members were included only if specific findings pertained also to nurses. As a consequence, Medical Subject Headings [MeSH] and free-text words used were: ‘*Antimicrobial Stewardship*’, ‘*Nursing*’, ‘*Attitudes*’, ‘*Perceptions*’, ‘*Role*’, ‘*Believes*’, ‘*Knowledge*’, and ‘*Behaviors*’. All these terms have been combined into the search strings with the Boolean Operator ‘AND’. Electronic database searches were completed on January 25th, 2022.

## Screening and study selection

**Fig. 1:**
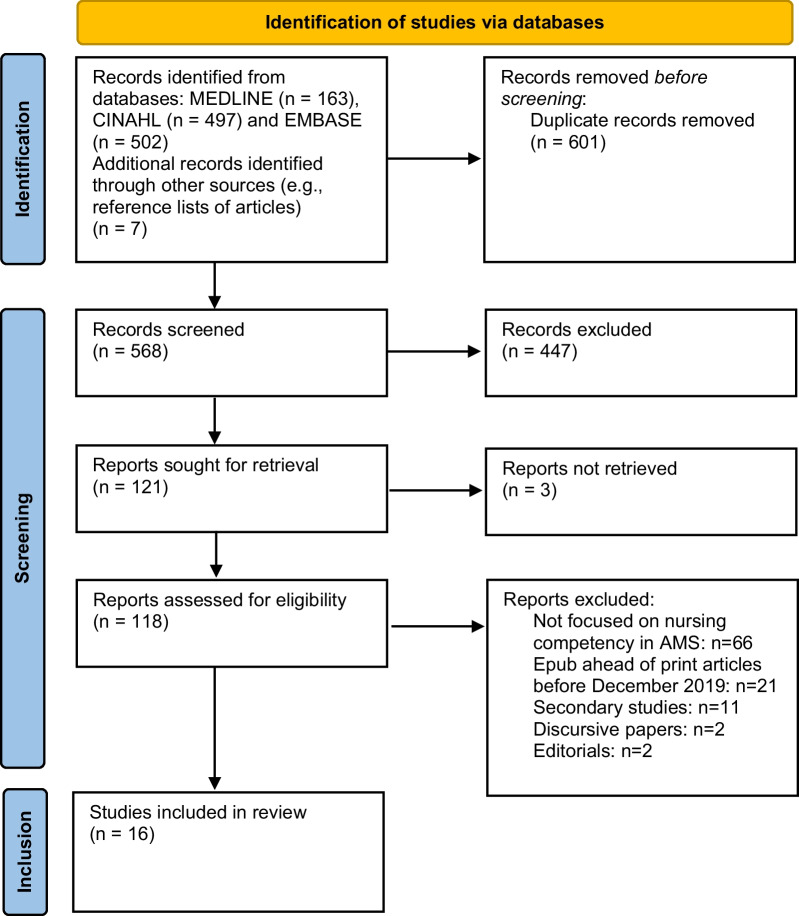
Review flow diagram

The screening of titles and abstracts was performed by three researchers (MD, TBW, DR) to identify articles’ eligibility in relation to the inclusion criteria. Then, an independent full-text evaluation was performed by the same researchers to determine if studies fully met the inclusion criteria. When disagreements occurred (e.g., nurses not recognizable among the multidisciplinary team members in the sample), the final decision to include or exclude an article was made by consensus. As reported in the PRISMA flow diagram (Fig. 1), the flow of study inclusion is summarized, together with the reasons for exclusion. At the end of the process, 16 papers were retrieved.

## Data extraction

Data were extracted in a Microsoft Excel® spreadsheet. The following data were extracted from each selected study and reported in the grid: (a) author(s), journal, publication year and country; (b) study design, setting (e.g., acute hospital), aims and participants’ profile; (c) item(s) under study (e.g., nurses’ performance of antibiotic stewardship activities); (d) metrics used (e.g., a web-based questionnaire); and (d) key findings. The full grid is available as Table 1.

**Table 1:** Data extraction process: steps performed

First author, year and country	Design	Setting	Aim(s)	Participants’ profile	Item(s) under study	Metrics	Key findings
*Quantitative descriptive studies*							
Akbar et al. [29] Saudi Arabia (Asia)	Cross-sectional study	Three programs of the university: clinical laboratory science, nursing, and pharmacy	(i) evaluate the knowledge of future healthcare workers in Saudi Arabia on antibiotics, antibiotic use, and antibiotic resistance; (ii) determine the factors influencing students’ knowledge	HCPs students. 284 clinical laboratory science, nursing, and pharmacy students; mean age was 21.64 (SD = 2.20) years. Among students, 53.9% were male and 52.5% urban dwellers. More than half of the samples comprised nursing students (51.4%), and 28.2% and 20.4% of the respondents were pharmacy and CLS students, respectively. The highest proportion of the sample was sophomores (36.6%), whereas the lowest was seniors (27.8%)	Students’ knowledge of (1) antibiotics, (2) antibiotic use, (3) antibiotic resistance	A questionnaire was used to collect data for the study variables. Ten, five and eight questions assessing students’ knowledge of antibiotics, antibiotic use, and antibiotic resistance (with a 4-point Likert scale measuring the relevance of the items)	The study found that students have above-average knowledge of antibiotics and antibiotic resistance, whereas their knowledge of antibiotic use was inadequate. Several factors, including gender, program, academic level, awareness about antibiotic resistance, attendance to seminars/training, and belief on the seriousness of antibiotic resistance problem, affect students’ knowledge. The findings suggest that students’ knowledge in these areas should be improved
Ashiru-Oredope et al. [33] UK [Multiple European countries] (Europe)	Cross-sectional study	A Project Advisory Group comprising 87 individuals representing 30 EU/EEA countries and European professional organizations	(i) assess knowledge, attitudes, and behaviors of healthcare workers in 30 EU/EEA countries with respect to antibiotics, antibiotic use, and antibiotic resistance; (ii) provide a baseline dataset to design and evaluate future policy, communication, and educational interventions; (iii) support the evaluation of awareness raising campaigns including European Antibiotic Awareness Day	HCPs. 18,365 healthcare workers (7,351 medical doctors, 4,312 nurses, 3,258 pharmacists, 1,085 dentists, 633 allied health professionals) 97% were over the age of 25 years and 70% were women. The respondents predominantly practiced in hospitals (49%), the community (22%), or in pharmacies (10%)	The COM-B (capability, opportunity, motivation and behavior) model (1) Antibiotic use and antibiotic resistance-information available on antibiotic use and antibiotic resistance; (2) Campaign and training; (3) Prescriber questions; (4) Undergraduate health student questions	43-item web-based questionnaire (multiple choice questions, statements testing knowledge using a true or false answer, and statements assessing attitudes and behaviors by seeking agreement using a 5-point Likert scale)	Knowledge of antibiotics and their use was higher (97%) than knowledge of development and spread of antibiotic resistance (75%). 60% of respondents stated they had received information on avoiding unnecessary prescribing, administering or dispensing of antibiotics. Among respondents who prescribed, administered or dispensed antibiotics, 55% had provided advice on prudent antibiotic use or management of infections to patients, but only 17% had given resources. For community and hospital prescribers, fear of patient deterioration or complications was the most frequent reason (43%) for prescribing antibiotics that were considered unnecessary. Community prescribers were almost twice as likely as hospital prescribers to prescribe antibiotics due to time constraints or to maintain patient relationships
Barchitta et al. [34] Italy (Europe)	A clustering analysis from a cross-sectional study	Multi-country and multi-professional survey launched by the European Centre for Disease Prevention and Control	Compare knowledge, attitudes and behaviors on antibiotic use and resistance across different groups of Italian HCW	HCPs. 2,167 healthcare workers; 479 medical doctors (both physicians and surgeons), 354 nurses and midwives, 140 pharmacists (both community and hospital pharmacists), 97 dentists, and 215 other HCW (i.e. hospital managers, pharmacy technicians, physiotherapists, biomedical scientists, and allied health professionals)	Knowledge, attitude and behaviors of HCW on antibiotics, antibiotic use and resistance	Online survey tool to evaluate capabilities, opportunities and motivations that enable prudent behavior on antibiotic use (see Ashiru-Oredope et al. [34])	Italian HCW exhibited different knowledge, attitudes, and behaviors on antibiotic use and resistance. These findings raised the need for educational and training interventions that target specific professional groups
Bouchoucha et al. [25] Victoria (Australia)	Cross-sectional study	Bachelor of Nursing and other combined nursing degrees at a University in Victoria, Australia	Elicit nursing students’ perspectives and perceptions of the nurse’s role in antimicrobial stewardship	Nursing students. 321 nursing students enrolled in an Australian university 291 (91%) were female and 30 (9%) were male with a mean age of 25.7 years (SD = 8.44). Of the participants, 112 (61%) had no experience working in healthcare, 40 (12%) were enrolled nurses and 31 (9.7%) were registered nurses from overseas. The majority (83.2%) of participants were domestic students	Knowledge and opinions regarding the nurse’s role in AMS and on a range of topics relating to antimicrobial stewardship and antimicrobial resistance	Series of closed and open-ended questions	Findings underscore the need to engage nursing students in discussions that explore the problem of antimicrobial resistance and the important role nurses play in Antimicrobial Stewardship programs
Carter et al. [21] Pennsylvania [multiple regions of the States] (USA)	Cross-sectional study	Acute care hospitals that are members of the Association of Professionals in Infection Control and Epidemiology	Describe clinical nurse involvement in antibiotic stewardship programs (ASPs)	RNs. 207 infection preventionists (IPs) working in acute care hospitals	(1) Leadership support of nurses’ involvement in antibiotic stewardship; (2) Nurses’ performance in antibiotic stewardship activities; (3) Nurses’ knowledge and training in antibiotic stewardship	The survey consisted of 40 multiple-choice and free-text questions	Study findings indicate the need for nurse leaders to improve the preparation and integration of clinical nurses in ASPs. While clinical nurses routinely perform activities that contribute to optimal antibiotic use, the knowledge and competency of clinical nurses in these activities and their formal integration in ASPs are minimal
Hamilton et al. [22] Utah (USA)	Cross-sectional study	The 2018 annual American Association of Nurse Practitioners (AANP) conference in Denver, Colorado	Describe the knowledge, attitudes, and perceptions of NPs about antibiotic use, resistance, and stewardship	NPs. 194 NPs completed the questionnaire. 88% were female, and 12% were male. The majority of the respondents (70%) had a master’s degree, and the range of experience was 0–45 years (mean 11 years). Family NP was reported by 65% as the population for their initial NP education, with 12% indicating Adult-Gerontology Primary Care NP and 9% Adult Acute Care NP. 23% of respondents described their current health care setting as Private Office, 18% as Hospital/Acute Care, and 29% as Community	(1) Attitudes that influence decisions; (2) Attitudes toward empiric selection; (3) Perceptions about antibiotic use and resistance; (4) Perceptions toward educational resources	The questionnaire used in this study consisted of 54 items. Attitudes that influence decisions about antibiotics and empiric selection were scored on a 1–5 scale (never, rarely, sometimes, often, and always). The perception questions about antibiotic use and resistance were scored on a 5-point Likert response scale, from “strongly agree” to “strongly disagree.” Education resources were scored on a 5-point scale, from “very useful” to “never useful”	Factors affecting the decisions of antibiotic prescriptions included patient condition (79%) and patient cost (58%). Nurse practitioners based their antibiotic decisions on the antibiogram (63%) in their setting, whereas 56% indicated they start with broad spectrum and tailor antibiotic choices after cultures are received. Nurse practitioners understood that inappropriate antibiotic use causes resistance (97%), harms the patient (97%), and optimum antibiotic use will reduce resistance (94%). Participants also recognized that strong knowledge of antibiotics was important (94%) and felt confident in using antibiotics (86%). However, 94% agreed that antibiotics are overused nationally, and only 62% thought antibiotics were overused in their setting
Herawati et al. [30] Indonesia (Asia)	Observational descriptive study with a cross-sectional design	3 hospitals in the East Java province, Indonesia	Identify the differences among health care professionals’ knowledge and beliefs about antibiotic stewardship	HCPs. The professions included are doctors, pharmacists, midwives, the AMS team members, and nurses. The excluded professions are psychiatrists, radiologists, obstetric and neonatal nurses, and hemodialysis nurses. (65 nurses, 61 midwives, 27 pharmacists, 45 pharmacy technicians, and 59 physicians)	Healthcare professionals’ knowledge and belief (perceived threat, perceived self-efficacy, perceived benefit, and perceived barrier) on antibiotic stewardship to be able to design training that meets their need	The questionnaire consisted of 43 questions: 12 questions were used to assess knowledge, and 31 questions to assess belief. The 31 belief questions consisted of 10 questions to assess perceived threats, 11 questions to assess perceived self-efficacy, 8 questions to assess perceived benefits, and 2 questions to assess perceived barrier	Among healthcare professionals, knowledge and belief differences of antibiotic stewardship vary widely. Antibiotic knowledge is associated with positive belief and behavior that contribute to the adherence to a judicious use of antibiotics and reduce antibiotic utilization. The Health Belief Model (HBM) theory presumes several constructs (perceived severity, perceived benefit, self-efficacy, cues of action) to predict behavior
Lim et al. [31] Singapore (Asia)	Cross sectional survey	acute care tertiary public hospital	Evaluate nurses’ knowledge and perceptions of antimicrobial stewardship	RNs. 241 nurses with enrolled or registered nursing licenses, working in medical, surgical, critical care areas, high-dependency, and isolation wards. About half of the participants were below 30 years old (55.6%); the majority were female (84.6%) and staff nurses (81.3%), while just over half had a clinical experience of 7 years and above (51.4%). Most of the participants were working in general medical and surgical wards (85.5%)	Assess participants’ awareness of the term AMS, level of knowledge, and perception of nurses’ role in AMS, as well as the need for measures to minimize the impact of AMR and AMS to be included in the undergraduate nursing curricula	An online 13-item questionnaire that explored the nurses’ attitudes and knowledge of AMS was used in this study with permission. The questionnaire consisted of ‘yes’ or ‘no’ questions and Likert scale questions, ranging from ‘1’ (none) to ’5’ (excellent).	Improving the awareness of and education on AMS and AMR among nurses is an essential element in infection and prevention control. Nurses’ role can be further strengthened by integrating fundamental training on AMS in the undergraduate nursing curriculum and providing continuing education in the clinical setting, as well as promoting the role of nurses in advocating for patients and AMS
Monsees et al. [23] Missouri (USA)	Cross-sectional survey	Nine hospitals ranged in size from 42 to 562 beds serving pediatric and adult populations in 2 different metropolitan areas	Determine bedside nurses’ recognition and performance confidence in AMS	RNs. A total of 4,282 direct care RN and licensed practical nurses were asked to participate but only 558 study subjects completed the survey. Highest number of respondents were nurses who graduated within1-5 years (n = 190, 34.1%), with the second highest category representing nurses with more than 15 years of experience (n = 176, 31.5%)	General AMS questions were included to bolster understanding of how nurses perceive their participation, knowledge, and role as antibiotic stewards. Using free text, respondents were also asked to identify strategies to enhance nurse engagement or elaborate on their role	The final survey had 48 questions- 9 demographic, 11 general AMS, 10 practice, 10 confidence, and a total of 8 from the Agency of Healthcare Research and Quality Hospital Survey on Patient Safety Culture (public domain), including 5 on teamwork and 3 on communication openness. All items were scored on a 5-point Likert scale	Nurses identified a professional role in AMS processes, even though safety culture inhibited their involvement. These findings can help enhance the inclusion of nurses in AMS efforts
Padigos et al. [26] Auckland region (New Zealand)	A cross-sectional survey design	The greater Auckland region of New Zealand	Investigate knowledge of registered nurses (RNs) on antibiotics, AMR and their understanding of AMS	RNs. Two hundred ninety-eight (n = 298) respondents from diverse nursing backgrounds completed the survey. Most respondents were trained in New Zealand and had more than 5 to 10 years of nursing experience. The median age group was 40–49 years old. More than half (161/298, 54%) had post graduate degrees	The views of RNs on their potential roles in AMS, their knowledge, understanding of AMR and use of antibiotics. The intended outcome was to determine nurses’ gaps in knowledge related to antimicrobials and AMR	A56-item online questionnaire was developed based on existing literature that related to antibiotic use, AMR and AMS principles. In addition, specific questions were also taken from a questionnaire that was previously used in measuring knowledge of AMR in patients and nurses	Nurses play an essential role in promoting AMS practices. However, a good understanding of antibiotics, AMR and AMS is needed to effectively embed these concepts in clinical practice. Hence, addressing these educational needs is of paramount importance
*Qualitative studies*							
Dowson et al. [27] Victoria (Australia)	One-on-one, semi-structured, qualitative interviews	Health professionals (i.e., nurses, general practitioners and pharmacists) working in aged-care homes in Victoria, Australia	(i) Describe health professionals’ perspectives on antimicrobial use near the end of life in aged-care homes. (ii) Investigate the potential opportunities for nurses to undertake antimicrobial stewardship activities near the end of life in aged-care homes	RNs. Twelve nurses, six general practitioners and two pharmacists providing routine care to residents of aged-care homes were interviewed. Diversity in terms of years of experience, aged-care funding type (government, private-for-profits and not-for-profits) and location metropolitan and regional) were sought	1. What is the environmental context and social basis of nurse participation in antimicrobial prescribing near the end of life in aged-care homes? 2. What does this assume implicitly or explicitly about aged-care nurses and their workplace relationships? 3. Of what larger process is this behavior a part? 4. What are the implications of such nurse behaviors for residents, general practitioners and families in aged-care homes with regards to antimicrobial prescribing near the end of life?	Participants were approached for one-to-one interview, after which no further contact was initiated by the researchers. A participant demographic questionnaire recording the participant’s age, profession, years in the profession, and years with their current aged-care home was facilitated by the interviewer. Demographic details about the aged care homes where participants were employed (or serviced) were also recorded by the interviewer. For participants who agreed to be audio recorded, the recorder was turned on after completion of the demographic questionnaire. The interviews followed a pre-set semi-structured interview guide, with early interviews and data analysis informing exploration in later interviews. The semi-structure interview guide was pilot tested with an experienced aged-care nurse and revised once prior to the start of the interviews on the basis of that pilot testing	Nurses have important roles in facilitating advance care planning, care coordination, care delivery and communication with families and medical professionals; these duties present important opportunities for nurses to lead antimicrobial stewardship activities appropriate for care near the end of life in aged-care homes
Kirby et al. [28] New South Wales (Australia)	Qualitative one-on-one, semi structured interviews	Four hospitals (three public and one private), across metropolitan, regional and remote areas, in two Australian states	Explore Australian hospital nurses’ views on antimicrobial resistance and AMS in a hospital setting, in order to better understand the opportunities for and challenges to integration of nursing staff in antimicrobial optimization within hospital settings	RNs. 86 nurses (77 females, 9 males), from a range of hospital departments, at a range of career stages	(1) Accounts of the significance of AMR (Antimicrobial resistance) and use in everyday nursing work, (2) The nursing role in antimicrobial decisions, inter-professional dynamics around antimicrobial use. (3) Nurse’s experiences and knowledge of AMS in the hospital context	Semi structured interviews, lasting between 20 and 60 min, were conducted by four research team members (three females, one male; all university-based sociologists from Anglo-Australian backgrounds, experienced in qualitative interviewing) between 2014 and 2019. Participating nurses worked within a range of departments and had various levels of experience and seniority (Table 1 includes detailed participant characteristics). Following written informed consent, interviews were digitally audio recorded and transcribed in full	As frontline staff in inpatient care, nursing engagement in AMS presents considerable yet currently underused opportunities. Nursing identity is well aligned with stewardship identity, and thus significant missed opportunities currently exist for nurses to contribute to practice, education, and research and policy efforts to reduce AMR. These missed opportunities exist largely because of interprofessional power imbalances within the social world of the hospital. Expanding AMS to better empower nurses is crucial to ongoing attempts to curb the threat of AMR by improving judicious use of antimicrobials
Rout et al. [35] South Africa (Africa)	Qualitative research	This study was conducted in a 20-bed adult ICU/high-care unit in a 200-bed private hospital in KwaZulu-Natal, South Africa (SA), which admits medical and surgical patients managed by non-intensivists	Explore the views of healthcare professionals regarding barriers to the antimicrobial stewardship role of the nurse in intensive care	RNs. Fifteen participants from the disciplines of nursing, surgery, anesthetics, internal medicine, microbiology, and pharmacy in a general intensive care unit. Nursing participants included two nurses from hospital management who had set up the AMS program two years previously, and six clinical ICU nurses whose responsibilities as shift leaders included daily AMS rounds. These were registered professional nurses; one held an additional qualification as an ICU nurse; the remaining five had experience of working in this area of nursing but held no specialist qualification. Non-nursing participants included a microbiologist, pharmacist, two anesthetists, two physicians and two surgeons. The microbiologist was representative of one of the private laboratories that served the study hospital and the pharmacist was a representative from the hospital pharmacy. The clinicians were specialists who had admission rights in the ICU	Barriers of AMS role of nurses in intensive care	Semi-structured individual interviews with all participants	The nursing role within antimicrobial stewardship was negatively affected by both staffing and collaborative difficulties, which impacted on the implementation of antimicrobial stewardship within the unit
van Gulik et al. [32] Thailand (Asia)	Qualitative descriptive study	A 1000-bed university hospital in Bangkok, Thailand	Explore how organizational multidisciplinary leaders and clinical nurses perceive nurses’ roles in AMS	RNs. A sample of 33 participants made up of organizational leaders and nurses. The 15 organizational leaders interviewed were: health service Director, Director of Nursing, Director of Pharmacy, Chair of the Infection Prevention and Control (IPC) committee, IPC nurse manager, infection control specialists, surgeons, an infectious diseases physician, operating suite nurse manager, intensive care unit nurse manager, AMS and clinical pharmacists and the Virology Department head scientist	(1) Current governance, educational and practice context. (2) Nurses’ current role in AMS in hospital. (3) Barriers to nurses’ engaging in AMS Education, knowledge and support, workload, collaboration and communication	Individual and focus group interviews. The topic guide included two open ended questions: “Can you talk about nurses’ current role in AMS in this hospital?” and “What do you think are some of the barriers to nurses’ engaging in AMS? “	Nurses currently participate in AMS by supporting system processes, monitoring safety and optimal antibiotic use, and patient education. A lack of clear articulation of nurses’ role and traditional professional hierarchies limits active participation. Inconsistent engagement was perceived as due to a failure to prioritize AMS activities, a lack of formal policies, and a need for further education
*Mixed-methods studies*							
Knobloch et al. [24] Wisconsin (USA)	Mixed methods study	Interviews were conducted at six community-based outpatient clinics located in rural areas of two states. All three focus groups were conducted at the Madison Veterans Administration facility	i) Identify barriers and facilitators to guide concordant prescribing among NP prescribers in a Veterans Health Administration (VHA) outpatient setting; (ii) Explore perspectives about perceived roles in antibiotic stewardship efforts, both NPs and patient roles	NPs. 14 NPs for an in-person interview; two interviews conducted by phone due to NP preference. A total of 15 Veterans (10 male, 5 female) participated in the focus groups	(1) People involved in the process of prescribing; (2) tasks: diagnosing, guideline retrieval, and prescribing; (3) tools: electronic medical record, guidelines, and testing equipment; (4) organization; policies related to antibiotic prescribing and leadership aspects of implementing stewardship activities; (5) Environment; physical environment of the ambulatory clinics	The Systems Engineering Initiative for Patient Safety (SEIPS) model was used. Face-to-face semi structured interviews were carried out. The interviews covered topics such as appropriate prescribing, perceptions and ideas about the NP role in antibiotic stewardship, participating in developing an intervention targeting NPs, the veteran’s policy mandating antibiotic stewardship, and barriers and facilitators to concordant prescribing in the outpatient VA setting. There were three focus groups. Questions from an existing health care-associated infection patient engagement group, which included Veterans, were used. Quantitative data from over three calendar years (2017–2019) on NP prescribing for diagnoses categories were analyzed	Nurse practitioners reported satisfaction with resources, including ready access to pharmacists and infectious disease specialists. Building patient trust was reported as essential to prescribing confidence level. Veterans indicated the need to better understand differences between viral and bacterial infections. NP prescribing patterns revealed a decline in antibiotics prescribed for upper respiratory illnesses over a 3-year period
Mula et al. [36] Malawi (Africa)	Descriptive qualitative study	A referral hospital which is the largest teaching hospital, with a bed capacity of 1,200 serving a population of approximately 5.5 million. It provides tertiary care to the population in the south west districts of the country and serves an urban population of 920,000	Explore nurses’ role in antibiotic stewardship and the challenges they face in order to provide empirical evidence in support of ways to involve nurses	HCPs. Participants were senior doctors (n = 6), junior doctors (n = 6), pharmacists and laboratory technologists (n = 8, 4 from each discipline) Staffing level was cushioned by locum nurses who come from other wards, from retirement, or who were waiting to be employed	Three key events where antibiotic stewardship nursing activities most commonly take place were purposively selected for their data richness, namely: nursing handover report, which is a nursing care communication that routinely takes place at the end of each shift; night and day in order to ensure continuity of nursing care, antibiotic preparation event and ward rounds. Participants were encouraged to explain, based on their experience working with nurses, the roles of nurses and the challenges they face in antibiotic stewardship	The introductory question asked participants to describe their perceptions of antimicrobial resistance and antibiotic stewardship. Subsequent questions explored participants’ experiences and perceptions of nurse involvement in antibiotic stewardship interventions and perceived challenges	As to nurses’ roles, the main theme that emerged was that nurses have multiple antibiotic stewardship roles. The subthemes were: Nurses have a facilitating role in microbiology specimen investigations; Nurses have a role in contributing to prescription decisions; Nurses have a role to ensure antibiotics are available at point of care. Three main themes that resulted from the challenges were competency gaps, multidisciplinary team work challenges and limited resources

*AMS* antimicrobial stewardship, *AMR* antimicrobial resistance, *HCPs* healthcare professionals, *HCW* healthcare workers, *RNs* registered nurses, *NPs* nurse practitioners, *USA* United States of America

## Risk-of-bias assessment

The review team shared in advance the decisions about inclusion and exclusion to prevent information and selection bias. In addition, to ensure consistency of results, the following methodological requirements [16] were respected: (a) at least one major scientific database was accessed (= three database: MEDLINE, CINAHL, EMBASE); (b) verification of the study selection and data extraction were performed by at least two reviewers (= three reviewers [MD, TBW, DR]); (c) the narrative synthesis and the summary table were provided and checked by an additional independent researcher (= a fourth researcher was involved [MM, expert in AMS]). Lastly, quality appraisal of studies was performed. The methodological quality of quantitative, qualitative and mixed-methods studies was independently assessed by three of the authors (MD, TBW, DR) by adopting the Mixed Methods Appraisal Tool (MMAT) developed by Hong et al. [17] (Table 2). The methodological quality score was not a reason to exclude studies.

**Table 2:** Summary of mixed methods appraisal tool (MMAT) methodological quality assessment

Quantitative descriptive studies	Approach	MMAT criteria for quantitative descriptive studies				
		Is the sampling strategy relevant to address the research question?	Is the sample representative of the target population?	Are the measurements appropriate?	Is the risk of nonresponse bias low?	Is the statistical analysis appropriate to answer the research question?
Akbar et al. [29]	Survey	Y	Y	Y	N	Y
Ashiru-Oredope et al. [33]	Survey	Y	Y	Y	Y	Y
Barchitta et al. [34]	Survey	Y	Y	Y	Y	Y
Bouchoucha et al. [25]	Survey	Y	Y	Y	U	Y
Carter et al. [21]	Survey	Y	U	Y	N	Y
Hamilton et al. [22]	Survey	Y	U	Y	N	Y
Herawati et al. [30]	Survey	Y	U	Y	N	Y
Lim et al. [31]	Survey	Y	U	Y	N	Y
Monsees et al. [23]	Survey	Y	U	Y	U	Y
Padigos et al. [26]	Survey	Y	U	Y	U	Y

Qualitative studies	Approach	MMAT criteria for qualitative studies				
		IIs the qualitative approach appropriate to answer the research question?	Are the qualitative data collection methods adequate to address the research question?	Are the findings adequately derived from the data?	Is the interpretation of results sufficiently substantiated by data?	Is there coherence between qualitative data sources, collection, analysis and interpretation?
Dowson et al. [27]	Interview	Y	Y	Y	Y	Y
Kirby et al. [28]	Interview	Y	Y	Y	Y	Y
Rout et al. [35]	Interview	Y	Y	Y	Y	Y
Van Gulik et al. [32]	Focus group	Y	Y	Y	Y	Y

Mixed-methods studies	Approach	MMAT criteria for mixed-methods studies				
		Is there an adequate rationale for using a mixed methods design to address the research question?	Are the different components of the study effectively integrated to answer the research question?	Are the outputs of the integration of qualitative and quantitative components adequately interpreted?	Are divergences and inconsistencies between quantitative and qualitative results adequately addressed?	Do the different components of the study adhere to the quality criteria of each tradition of the methods involved?
Knobloch et al. [24]	Interviews, prescribing data analysis	Y	Y	Y	Y	Y
Mula et al. [36]	Multiple data source including focus groups, interviews, participants observations	Y	Y	Y	Y	Y

*Y* yes, addressed, *N* no, not satisfied, *U* unknown, cannot be determined

## Knowledge synthesis

Given the descriptive nature of the research question and the methodological heterogeneity of the included studies, data were narratively analyzed and categorized adopting an inductive thematic coding [18]. In particular, as first step, the dimensions of nursing competency under study were categorized according to their common organizational or clinical significance. Then, the second step consisted of identifying subthemes and themes. Categorization was performed according to (a) the Donabedian model dimensions, namely structure (e.g., setting, human resources), process (e.g., activities, co-operation) and outcomes (e.g., nursing sensitive outcomes) [19] and (b) the knowledge, attitudes and practice model, commonly used to explain how individual knowledge and attitudes can affect behavioral changes [20]. Each phase of this process was the result of the discussions among the authors.

## Results

### Study characteristics

Database searching returned 1169 articles, of which 568 were screened. Out of 118 articles assessed for eligibility, 16 studies met the inclusion criteria (Fig. 1). Reasons for full-text exclusion are given in Fig. 1. The specific characteristics of each study that met the inclusion criteria are presented in Table 1. Publications were mainly authored in the USA (n = 4, 25.0%; Missouri, Pennsylvania [multiple regions of the States], Utah, Wisconsin) [21–24], Australia and New Zealand (n = 4, 25.0%; Auckland, New South Wales, Victoria [two studies]) [25–28], and Asia (n = 4, 25.0%; Indonesia, Saudi Arabia, Singapore, Thailand) [29–32], followed by Europe (n = 2, 12.5%; Italy, UK [multiple European countries] [33, 34] and Africa (n = 2, 12.5%; Malawi, South Africa) [35, 36]. With regard to the research method, ten studies (62.5%) were quantitative in design, using surveys in all cases [21–23, 25, 26, 29–31, 33, 34]. Qualitative research included interviews (n = 3, 18.8%) [27, 28, 35] and focus groups (n = 1, 6.2%) [32]. The remaining two studies (12.5%) were mixed-methods in design, including multiple data sources (e.g., interviews, direct observations) [24, 36].

Table 2 displays the overall methodological quality of the included studies. The majority of them were good in quality clearly showing the methodology comprising sampling strategy, data collection and analysis.

## Dimensions affecting AMS competency

**Fig. 2:**
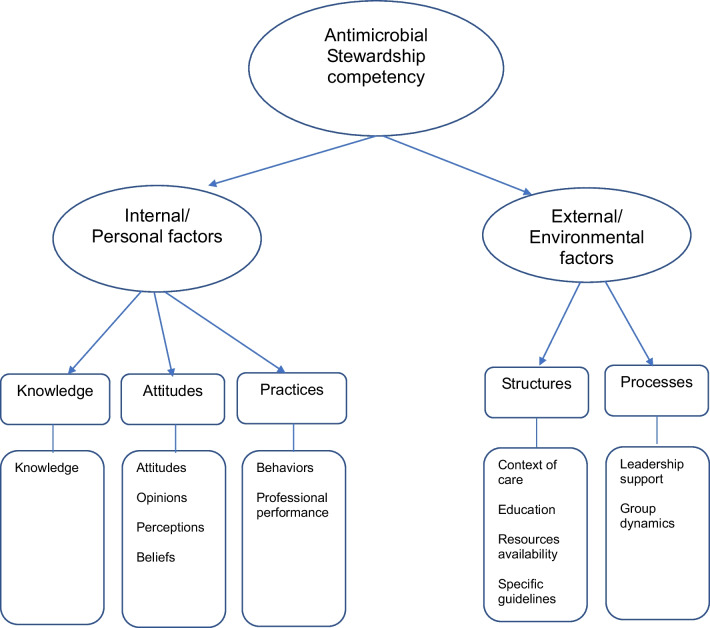
Dimensions affecting Antimicrobial Stewardship competency

Upon the analysis of the 16 selected studies, 13 dimensions, which were grouped in five subthemes and two themes, were identified (Fig. 2). An overview of all dimensions included in this review (dimension(s) under study, subthemes and themes) is presented in Table 3.

**Table 3:** Data synthesis by extracting and abstracting findings in common categories and themes

Abstraction: Themes	Abstraction: Subthemes	Dimension(s) under study		Studies
Internal/personal factors	Knowledge	Knowledge	Knowledge of AMS	Bouchoucha et al. [25]; Carter et al. [21]; Herawati et al. [30]; Kirby et al. [28]; Lim et al. [31]; Monsees et al. [23]
			Knowledge of antibiotic use	Akbar et al. [29]; Ashiru-Oredope et al. [33]; Barchitta et al. [34]; Padigos et al. [26]
			Knowledge of antibiotic resistance	Akbar et al. [29]; Ashiru-Oredope et al. [33]; Barchitta et al. [34]; Bouchoucha et al. [25]
			Knowledge of antibiotics	Akbar et al. [29]; Ashiru-Oredope et al. [33]; Barchitta et al. [34]
			Knowledge regarding the nurses’ role in AMS	Bouchoucha et al. [25]
			Significance of antibiotic resistance	Kirby et al. [28]
	Attitudes	Attitudes	Attitude with respect to antibiotics	Ashiru-Oredope et al. [33]; Barchitta et al. [34]
			Attitude with respect to antibiotic use	Ashiru-Oredope et al. [33]; Barchitta et al. [34]
			Attitude with respect to antibiotic resistance	Ashiru-Oredope et al. [33]; Barchitta et al. [34]
			Attitude in decision-making	Hamilton et al. [22]
			Attitude in antibiotic selection	Hamilton et al. [22]
		Opinions	Opinions regarding the nurses’ role in AMS	Bouchoucha et al. [25]
			Opinions regarding AMS	Bouchoucha et al. [25]
			Opinions regarding antibiotic resistance	Bouchoucha et al. [25]
			Views on potential nurses’ role in AMS	Padigos et al. [26]
		Perceptions	Perceived barriers of AMS	Herawati et al. [30]; Knobloch et al. [24]; Rout et al. [35]; van Gulik et al. [32]
			Perceptions about the nurses’ role in AMS	Knobloch et al. [24]; Lim et al. [31]; Monsees et al. [23]
			Perceptions about antibiotic use	Hamilton et al. [22]
			Perceptions about antibiotic resistance	Hamilton et al. [22]
			Perceptions toward educational resources	Hamilton et al. [22]
			Perceived threats	Herawati et al. [30]
			Perceived self-efficacy level	Herawati et al. [30]
			Perceived benefit of AMS	Herawati et al. [30]
		Beliefs	Belief in AMS	Herawati et al. [30]
			Awareness of AMS	Lim et al. [31]
	Practices	Behaviors	Behaviors concerning antibiotics	Ashiru-Oredope et al. [33]; Barchitta et al. [34]
			Behaviors concerning antibiotic use	Ashiru-Oredope et al. [33]; Barchitta et al. [34]
			Behaviors concerning antibiotic resistance	Ashiru-Oredope et al. [33]; Barchitta et al. [34]
		Professional performance	Experiences of AMS	Kirby et al. [28]; Mula et al. [36]
			Performance of AMS activities	Carter et al. [21]
External/environmental factors	Structures	Context of care	Circumstances in the aged-care situation and environment around antibiotic use	Dowson et al. [27]
			Practice context influence on AMS	van Gulik et al. [32]
			Physical environment influence on AMS	Knobloch et al. [24]
		Education	Educational influence on AMS	van Gulik et al. [32]
		Resources availability	Limited resources in AMS nursing role	Mula et al. [36]
		Specific guidelines	Guidelines/policies availability	Knobloch et al. [24]
	Processes	Leadership support	Leadership support of nurses’ involvement in AMS	Carter et al. [21]
			Governance influence on AMS	van Gulik et al. [32]
		Group dynamics	Interpersonal processes and dynamics around antibiotic use	Dowson et al. [27]; Kirby et al. [28]
			Multidisciplinary teamwork challenges in AMS nursing role	Mula et al. [36]

* AMS* antimicrobial stewardship, *AMS* antimicrobial stewardship

### Internal/personal factors

As to internal and personal factors, dimensions affecting AMS competency were measured at (a) knowledge, (b) attitudes and (c) practice levels. Factors at knowledge level were found to have the most influence on AMS competence (e.g., knowledge of antibiotics, knowledge regarding nurses’ role in AMS). Factors at attitudes level were instead studied with regards to attitudes (e.g., in antibiotic selection), opinions (e.g., regarding antibiotic resistance), perceptions (e.g., benefit of AMS) and beliefs (e.g., awareness in AMS). Factors referred to the practice were measured as behaviors (e.g., concerning antibiotic resistance) and professional performance (e.g., experiences of AMS).

### External/environmental factors

Studies evaluating the external and environmental factors included two subthemes namely (a) structures and (b) processes. Dimensions referred to the structures were evaluated as context of care (e.g., physical environment), education, resources and specific guidelines, and policies availability. Lastly, the processes were evaluated as leadership support (e.g., governance influence) and group dynamics (e.g., multidisciplinary teamwork challenges).

## Discussion

We identified 16 articles which related to nursing competency in AMS and were published in the last two years. The existence of such a large number of studies to our review reinforces the notion of an up-and-coming and meaningful interest on this particular research field. It is interesting to notice that these articles are spread across both low-income countries, such as Indonesia [30] and Malawi [36], and high-income states, such as Italy [34] and Missouri [23], which indicates that AMR is a contemporary global health threat. Moreover, it should be noted that qualitative research, which is more demanding than survey research, is limited; however qualitative studies is better methods to reveal facilitators and barriers to AMS implementation as they explore nurses’ perceptions of their experiences and roles within clinical settings in-depth [37].

The majority of the research examined in this review emphasized the significant contribution that nurses provide to AMS in hospital and community settings, where a high level of awareness among healthcare professionals has been documented [e.g., 31]. Healthcare students also proved to be acquainted with antibiotics and the related resistance, however their knowledge about the appropriate use was limited [29]. An international expert panel has recently developed a framework that addresses AMS to guide undergraduate nursing education (e.g., infection prevention and control) [38]. Several factors, such as the awareness of antibiotic resistance, the level of education, and beliefs, could influence students’ knowledge. It is important to engage healthcare students in communication and staff negotiations which address the issue of AMR with the aim of cultivating their knowledge, even in their earliest years of the undergraduate degree [25]. Moreover, the potential to prepare nurses for competencies and actions related to AMS is also ideal during university [38]. Improving the awareness of and the education on AMS and AMR also among registered nurses is an essential component in infection and prevention control. Nurses’ competency can be further strengthened by integrating fundamental education on AMS in the post-graduate studies and by providing continuing education in the clinical settings to enhance patient outcomes [31].

When looking at European level, healthcare workers’ knowledge of antibiotics and their use was higher (97%) when compared to their knowledge of the development and spread of antibiotic resistance (75%) [33]. The main reason for prescribing antibiotics was the fear of patient deterioration or the onset of complications; moreover, community prescribers were almost twice as likely as hospital prescribers to suggest antibiotics to deal with time constraints or to maintain their relationships with the patients [33]. Among healthcare professionals, differences in knowledge and beliefs about antimicrobial stewardship vary widely; there is a positive correlation between knowledge and behaviors [30]. Therefore, the use of the Health Belief Model (HBM) theory, which assumes several constructs (perceived severity, perceived benefit, self-efficacy, action signals) can support research to predict health workers’ behaviors [30]. Hamilton et al. highlighted that the factors that influence the decisions about prescribing antibiotic included patient condition (79%) and patient cost (58%) [22]. While 63% of nurse practitioners claimed to base their decisions on antibiotic use focusing on the antibiogram, 56% maintained to begin with broad spectrum antibiotics which are later customized upon the reception of cultures. Nurse practitioners are aware that resistance (97%), harmful effects on patient (97%), and resistance reduction due to optimum antibiotic use (94%) are the consequences of inappropriate antibiotic use. While 94% of nurse practitioners were well informed about the importance of having the proper knowledge of antibiotics and 86% felt certain about using them, 94% agreed on their overuse at national level and 62% on their overuse at work [22].

As to frontline staff in inpatient care, nursing engagement in AMS presents a considerable yet currently underused opportunity [35, 36]. Nursing identity is well aligned with stewardship identity, thus, at the moment, significant opportunities exist for nurses to provide their contribution to the practice, education, research and policy efforts intended to reduce AMR. These opportunities exist largely because of interprofessional and collaborative interplay. Expanding AMS to more empower nurses is pivotal to ongoing efforts to limit the trouble of AMR by perfecting judicious use of antimicrobials [28]. In spite of nurses being powerful contributors to AMS, the organizational culture can negatively impact on their ability to effectively provide their contribution. Strengthening team interaction can increase interdisciplinary participation in stewardship [23].

## Limitations

The primary limitation of our study is the lack of an univocal definition of nursing competency in AMS. Since the word “competency” has multiple interpretations, we avoided using it in search terms. In order to ensure the retrieval of studies having at least one dimension of nursing competency (e.g., knowledge, perceptions), a broad search-term strategy was utilized; however, it’s possible that not all relevant studies were found. Secondly, although there was a wide range of countries represented in this review, the number of included studies did not allow for conclusions concerning nursing competency in AMS in specific country contexts, especially in European countries, in which only two studies were found.

Moreover, regarding external and environmental factors, no study addressed also the outcome dimension. Most of the studies, which were included in this rapid review, referred to AMS in the hospital and community care; there were few studies taking place in long-term care facilities [27], where the incidence of antimicrobial resistance is as much as hospital resistance [39].

## Implications for practice and research

We conducted this rapid review to provide a map of dimensions on which scholars and practitioners should ponder when planning clinical governance, educational activities and research programs. At the clinical level, nurses have diversified roles in AMS. They have a leading position in facilitating investigations of microbiological samples, contributing to prescribing antibiotics and ensuring that antibiotics are available at the point of care. However, to provide the structure and drive the change, a committed leadership is required. Implementing AMR and AMS in the university curriculum, doubled by a thorough professional training at the post graduate level, is important in equipping nurses to fully act their role, resulting in a behavioral change and a more appropriate usage of antimicrobials. Then, further research is needed to address the gap in the theory guiding the identification and measurement of nurse competency regarding AMS. Shared job descriptions on AMS are required, in order to create hospital and community policies and measure the contribution of nursing care to the patients’ outcomes.

## Conclusion

The findings from this rapid review, mainly of surveys from observational studies, suggest that nursing competency in AMS seems to be influenced by a two-dimensional model, considering both internal/personal and external/environmental factors; the former consists in knowledge, attitudes and practice and at of the latter in structures and processes. However, there is little consensus regarding which competency dimensions are of importance to AMS and how these can be measured. Despite the emerging focus on the nurse’s role in AMS – which is well documented in the majority of the studies – there are still major challenges, such as defining specific and recognized competences, promoting multidisciplinary work, and coping with limited resources.

## Data Availability

Data sharing not applicable due to the nature of the study.

## Abbreviations

AMS: Antimicrobial Stewardship
AMR: Antimicrobial Resistance
WHO: World Health Organization
